# Comparison of catchment scale 3D and 2.5D modelling of soil organic carbon stocks in Jiangxi Province, PR China

**DOI:** 10.1371/journal.pone.0220881

**Published:** 2019-08-20

**Authors:** Tobias Rentschler, Philipp Gries, Thorsten Behrens, Helge Bruelheide, Peter Kühn, Steffen Seitz, Xuezheng Shi, Stefan Trogisch, Thomas Scholten, Karsten Schmidt

**Affiliations:** 1 Chair of Soil Science and Geomorphology, Department of Geosciences, University of Tübingen, Tübingen, Germany; 2 SFB 1070 ResourceCultures, University of Tübingen, Tübingen, Germany; 3 Department of Geobotany and Botanical Garden, Institute of Biology, Martin Luther University Halle-Wittenberg, Halle, Germany; 4 German Centre for Integrative Biodiversity Research (iDiv) Halle-Jena-Leipzig, Leipzig, Germany; 5 Institute of Soil Science, State Key Laboratory of Soil and Sustainable Agriculture, Chinese Academy of Sciences, Nanjing, China; The University of Sydney, AUSTRALIA

## Abstract

As limited resources, soils are the largest terrestrial sinks of organic carbon. In this respect, 3D modelling of soil organic carbon (SOC) offers substantial improvements in the understanding and assessment of the spatial distribution of SOC stocks. Previous three-dimensional SOC modelling approaches usually averaged each depth increment for multi-layer two-dimensional predictions. Therefore, these models are limited in their vertical resolution and thus in the interpretability of the soil as a volume as well as in the accuracy of the SOC stock predictions. So far, only few approaches used spatially modelled depth functions for SOC predictions. This study implemented and evaluated an approach that compared polynomial, logarithmic and exponential depth functions using non-linear machine learning techniques, i.e. multivariate adaptive regression splines, random forests and support vector machines to quantify SOC stocks spatially and depth-related in the context of biodiversity and ecosystem functioning research. The legacy datasets used for modelling include profile data for SOC and bulk density (BD), sampled at five depth increments (0-5, 5-10, 10-20, 20-30, 30-50 cm). The samples were taken in an experimental forest in the Chinese subtropics as part of the biodiversity and ecosystem functioning (BEF) China experiment. Here we compared the depth functions by means of the results of the different machine learning approaches obtained based on multi-layer 2D models as well as 3D models. The main findings were (i) that 3^rd^ degree polynomials provided the best results for SOC and BD (R^2^ = 0.99 and R^2^ = 0.98; RMSE = 0.36% and 0.07 g cm^-3^). However, they did not adequately describe the general asymptotic trend of SOC and BD. In this respect the exponential (SOC: R^2^ = 0.94; RMSE = 0.56%) and logarithmic (BD: R^2^ = 84; RMSE = 0.21 g cm^-3^) functions provided more reliable estimates. (ii) random forests with the exponential function for SOC correlated better with the corresponding 2.5D predictions (R^2^: 0.96 to 0.75), compared to the 3^rd^ degree polynomials (R^2^: 0.89 to 0.15) which support vector machines fitted best. We recommend not to use polynomial functions with sparsely sampled profiles, as they have many turning points and tend to overfit the data on a given profile. This may limit the spatial prediction capacities. Instead, less adaptive functions with a higher degree of generalisation such as exponential and logarithmic functions should be used to spatially map sparse vertical soil profile datasets. We conclude that spatial prediction of SOC using exponential depth functions, in conjunction with random forests is well suited for 3D SOC stock modelling, and provides much finer vertical resolutions compared to 2.5D approaches.

## Introduction

Soils are a fundamental part of ecosystem functioning and services [1]. As finite resources, soils contribute to food production, nutrient cycling, biodiversity and freshwater quality [2]. Furthermore, they are interconnected with other ecosystem functions and services, such as local and global climate alteration; and therefore, contribute indirectly to human well-being [3]. Among soil properties, soil organic carbon (SOC) plays an important role in this context. SOC increases the water-holding capacity (e.g. important for agriculture, forest and flood management), improves the physical properties of soils, such as nutrient availability for plants in agriculture and forestry, and accounts for carbon sequestration to mitigate climate change [4–6]. In forestry, there is strong interest in the effects of tree species and tree diversity on soil carbon input and mineralization as well as the net effects of these processes [7]. Knowledge about the interconnection between SOC, forests and the diversity of tree species as well as SOC stock degradation by soil erosion [8,9] and land cover change [10,11] can also help to implement countermeasures to reduce global warming [7]. Consequently, the implementation of a credible soil carbon auditing and monitoring to verify changes in SOC is crucial regarding soil security and carbon sequestration [7,12].

To preserve the functions and services provided by soils, a good quantitative understanding of the SOC stocks is required–both in the vertical domain of a soil profile as well as in the spatial domain over landscapes [13,14]. However, conventional soil maps use soil classes in horizontal dimension and soil horizons in vertical dimension. This categorical setup is often not precise enough and not well suited for interpreting soil functions and processes as well as for decision-making, since soil properties mostly vary continuous in space and time [15,16].

For the spatial prediction of continuous soil properties, such as SOC, methods of digital soil mapping (DSM) are suitable [17–19]. DSM is based on the soil forming factor concept [20] and the *scorpan* model introduced by McBratney et al. [21]. Both approaches illustrate soil information as a function of environmental covariates, influencing the process of soil formation. Terrain parameters, describing the shape of the land surface, are used widely as an environmental covariate in DSM. Terrain is an essential factor of soil formation and controls the effects of gravity, climate, lithology, water and biota [22–24]. Hence, models that are based on terrain parameters reproduce displacement and reallocation of soil (i.e. mass movements and soil erosion) and are of particular interest when modelling SOC at catchment scale [25]. Furthermore, terrain can not only be used to estimate or model soil displacement and reallocation, but also as a proxy for environmental covariates, which are not used as predictors, or inaccessible *scorpan*-factors. For instance, slope and aspect can serve as proxy for microclimate through its influence on local solar insulation [24]. The catchment area can serve as a proxy for soil fertility because of terrain driven water and SOC accumulation [19] and elevation, slope and aspect can act as proxy for parent material, tectonics and periglacial climate through strike and dip of the geological sediments and down-cutting processes [22,23,26].

For spatially modelling soil properties, different approaches have been established to derive relationships between soil properties and environmental covariates. However, for a reliable estimation of SOC stocks, the vertical dimension is crucial [13]. A common way of three-dimensional mapping is to consider the vertical dimension as multiple two-dimensional predictions, which can be interpreted in a three-dimensional way [17,27–29]. Because, multi-layered predictions do not provide full 3D soil information, since they are limited to the mapped depth increments. Information of the space between the mapped depth increments has to be derived on an interpretative and subjective basis. One approach is to vertically interpolate the single layers to construct a volumetric model, which is computationally intensive [30,31].

Therefore, multi-layered models are referred to as pseudo-3D mapping or 2.5D mapping [32]. To overcome these drawbacks, it is favourable to map soil properties as continuous depth function in the spatial domain [13,18], where the vertical distribution of soil properties is represented by depth functions, that are predicted spatially. These predictions allow the calculation of SOC stocks over the integral of the functions [33] as well as the calculation of fully three-dimensional maps at any vertical resolution [32,34–37].

Besides geostatistical frameworks [38,39], different depth functions have been applied for 3D modelling: power, logarithmic [32,40], exponential decay [32,33], polynomial [34,36] and equal-area spline functions [31,41].

While with 2.5D mapping soil properties are directly predicted at specific depth levels using the environmental covariates [17,29], 3D approaches use environmental covariates to predict parameters of the depth functions [34], which are abstract soil properties. According to the *scorpan* model, soil properties can be spatially mapped with neighbourhood relations solely [21], which also have been used for 3D modelling [36,40,42,43]. Over the past years, machine learning techniques have become a standard technique in DSM due to several advantages like dealing with non-linearity or the handling of large datasets. Aldana Jague et al. [33] used multiple linear regression (MLR) to model SOC incorporating terrain covariates, while Gasch et al. [43] compared spatial and terrain covariates using random forests (RF) and regression kriging for mapping SOC at different depth layers. Piikki et al. [27] used multivariate adaptive regression splines (MARS) to model clay and sand fractions as well as organic matter based on proximal soil sensing data. Several other studies also suggest that machine learning techniques, such as artificial neural networks (ANN; [41,44]), random forests (RF; [17]) and support vector machines (SVM; [45]), can be applied successfully in DSM.

The objectives of this study were to test the spatial prediction of four soil profile depth functions for modelling SOC content and bulk density with different machine learning methods based on multi-scale terrain covariates. The tested soil profile depth functions are polynomials of 2^nd^ and 3^rd^ degree, natural logarithmic and exponential functions. The machine learning methods used to model the depth functions spatially were multivariate adaptive regression splines (MARS), random forests (RF) and support vector machines (SVM) with radial basis functions. We validated the machine learning models with 10-fold cross-validation and evaluated the results of the 3D mapping approach by comparing it with the predictions of the more common multi-layered 2.5D modelling approach based on five layers.

## Material and methods

### Study area and sampling design

The BEF-China study sites are artificial biodiversity experiments on property leased and managed by the project partner Institute of Botany, Chinese Academy of Sciences, 20 Nanxincun, Xiangshan, Bejing, 100093, PR China. Field studies did not involve endangered or protected species and no specific permissions for field research were required.

**Fig 1:**
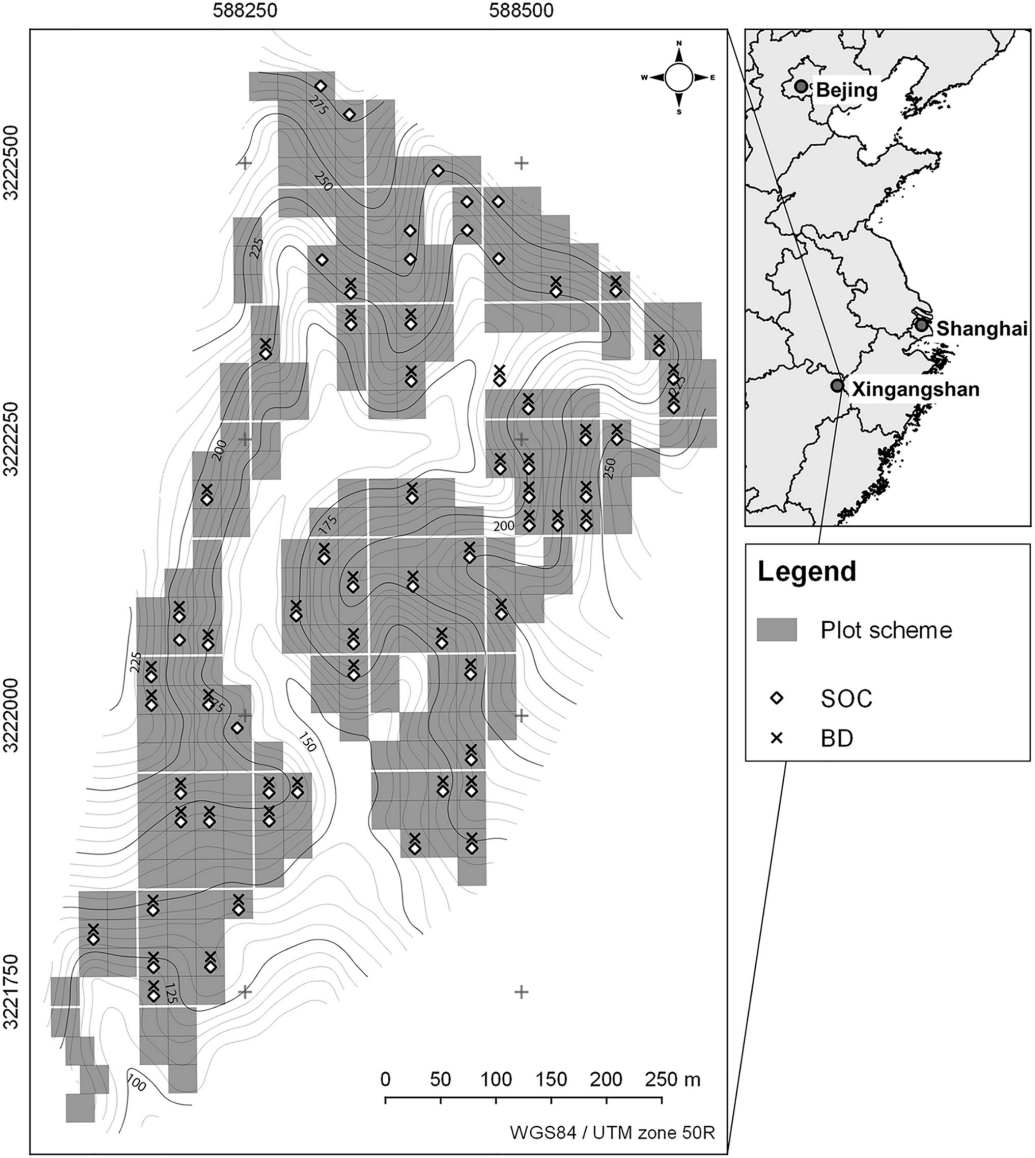
Study area in mainland China with BEF-China plot scheme and indication of sampled plots. Upper right panel with permission by R. Hijmans; https://gadm.org/.

The biodiversity and ecosystem functioning (BEF) China project [46] is located near Xingangshan, Jiangxi Province, PR China (UTM/WGS84: 50R 588000 3222000), about 400 km south-west of Shanghai (Fig 1). The study site is a topographically heterogeneous environment in a small catchment of 26.7 ha leased by the Institute of Botany of the Chinese Academy of Sciences (CAS). It features an elevation ranging from 105 to 275 m a.s.l., slopes inclined 29° in average and a maximum slope inclination of 45°, which are typically convex [19]. Non-calcareous slates with varying sand and silt content and grey-green sandstone constitute the bedrock. Predominant soil types are Endoleptic Cambisols with Anthrosols at the hillsides and Gleysols at the valley bottom. The mean soil depth is 0.6 m with underlaying isomorphic weathered slate (saprolite; [19]). Soil texture ranges from silt loam to silty clay loam [47]. The climate is typically subtropical with monsoons in summer, a mean annual temperature of about 17 °C and long-term average annual rainfall of about 1800 mm [48] but with a drier period from 2009 to 2012 [49].

About 18 ha were covered with 271 experimental plots. In total 8.7 ha at the valley bottom were not part of the experimental design due to paths and rivulets. Plots had a size of 25.8 m × 25.8 m (traditional Chinese unit of 1 mu, 1/15 ha) and were replanted in 2008 after clear-cut of a commercial Chinese fir plantation. One plot comprised 400 (20 × 20) trees in monocultures and mixtures of 2, 4, 8, 16 and 24 species. Species composition of the plots was based on random as well as non-random (plant trait-oriented) extinction scenarios, where all species were represented equally (broken-stick design). The datasets used in this study comprised soil samples from random subsets of all species and species richness levels referred to as VIPs (Very Intensively Studied Plots). For details on the experimental design, see Bruelheide et al. [46] and Trogisch et al. [50].

### Datasets

**Fig 2:**
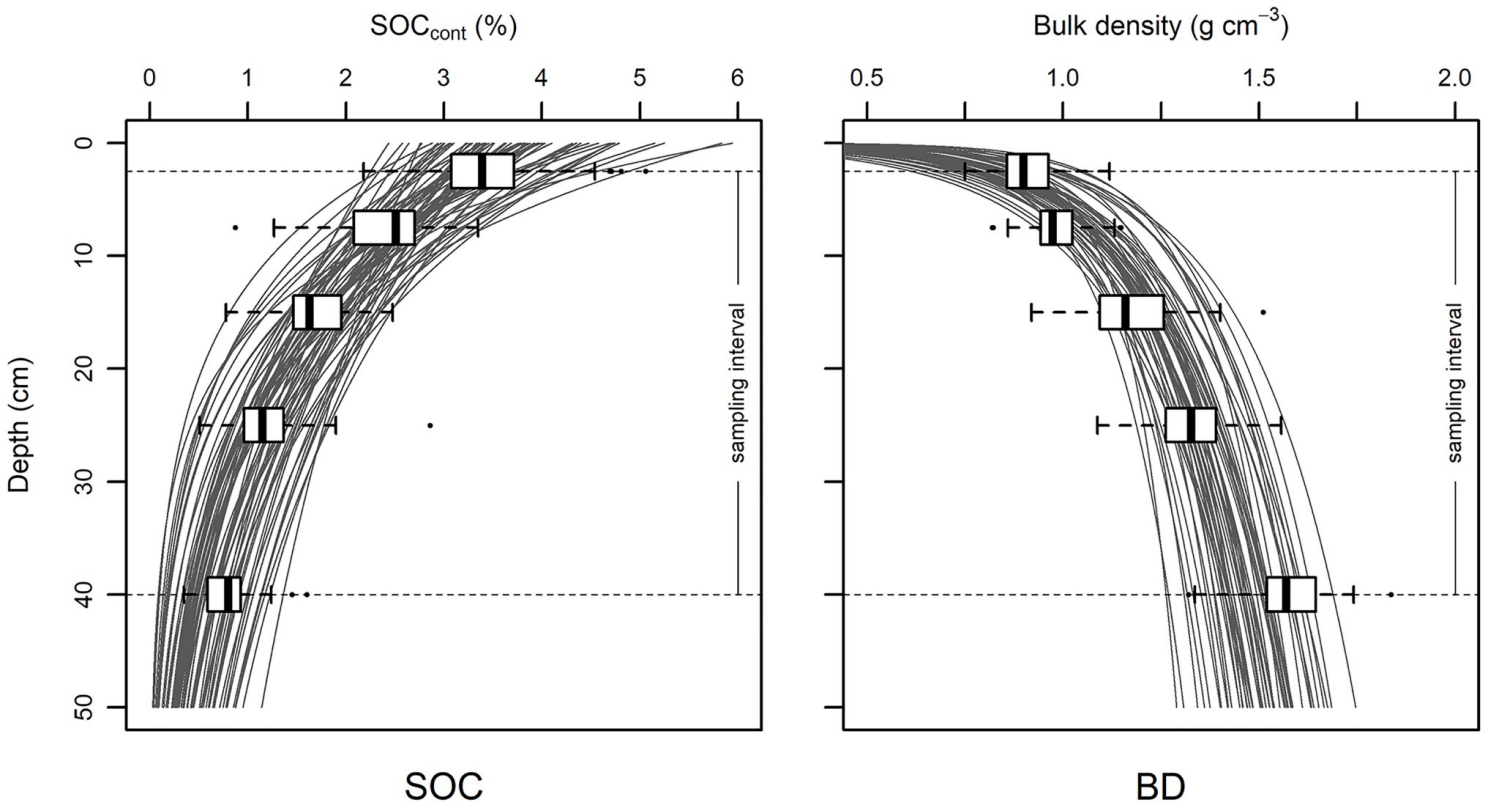
Datasets for SOC and BD used in this study summarized in boxplots. The boxplots show the variation of the SOC and BD values for each depth increment. SOC and BD samples were taken in five depth increments and 9 cores per plot were bulked (Note that depth increments do not increase linearly). The grey lines show model depth functions (3^rd^ degree polynomial for SOC and natural logarithmic function for BD; see subsection “3D mapping with soil depth functions”).

All described datasets are part of the legacy database of BEF-China. Soil sampling was conducted in 2014. Nine cores on a regular grid basis (3 cm in diameter) were taken at each of the 67 VIPs according to the BEF-China experimental design (Fig 1; [46]). The samples were bulked for each depth increment (0–5 cm, 5–10 cm, 10–20 cm, 20–30 cm and 30–50 cm) and were referred to as dataset SOC (n = 67; Fig 2). Fine roots and charcoal were sorted out manually. For dry combustion CNS-analysis, a Vario EL III (Elementar, Hanau, Germany) was used. Due to acidic soil conditions there was no detectable carbonate fraction, and thus total carbon represented SOC [19]. SOC content ranged from 5.06 to 0.35% decreasing with depth.

Bulk density samples (n = 55) were taken in April 2015 with soil sample rings (100 cm^3^) and five replicates for each depth increment at the VIPs. Bulk density was determined gravimetrically and was referred to as dataset BD (Fig 2). Bulk density ranged from 0.75 to 1.84 g cm^-3^ increasing with depth.

Since some plots with SOC samples did not have BD data (Fig 1), both soil properties were modelled individually instead of calculating and modelling the SOC stocks directly. This ‘model-then-calculate’ approach is a useful alternative to the ‘calculate-then-model’ approach. Both were compared by Orton et al. [51].

**Fig 3:**
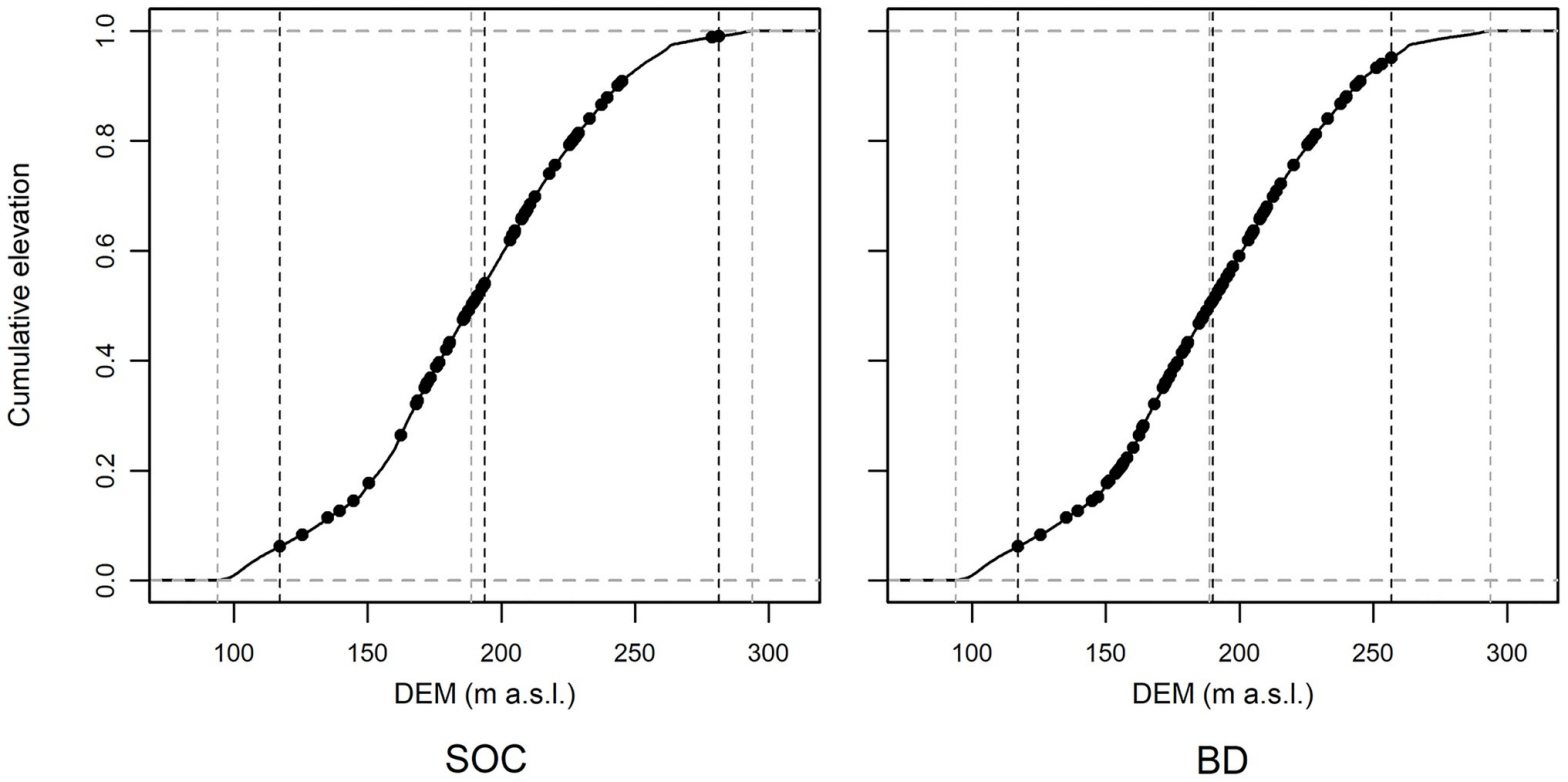
Empirical cumulative distribution functions (ECDF) for SOC and BD datasets. The ECDFs show the locations of the sampling sites in the state space of the elevation (DEM) in metres above sea level (m a.s.l.). The aim is to show the coverage of the DEM feature space by the samples. It can be seen that most samples are located in the mid-range of the elevation values. Therefore, predictions at grid locations which are only sparsely covered by the samples (i.e. locations close to the minimum and maximum values of the DEM) may be less accurate. The minimum, median and maximum values of both datasets (DEM and sampling locations) are shown with vertical lines (dashed grey: DEM, dashed black: sampling locations) to compare the full range of the respective feature spaces.

The digital elevation model (DEM) had a resolution of 5 m and was generated by ordinary kriging [52] based on differential global positioning system data (DGPS) with 1956 points (73 points per ha; [19]). The distribution of datasets SOC and BD over the DEM is shown in Fig 3. Dataset SOC covered the elevation data more comprehensively compared to the dataset BD.

### Digital terrain analysis

Environmental covariates that describe the morphometry of a landscape are grouped in four major classes of terrain attributes: local, regional, combined (i.e. combinations of local and regional) and solar morphometric variables. Given that many terrain attributes can be calculated based on different equations or modelling approaches and because it is unknown which version would be most suitable for modelling SOC and BD within the study area, we used multiple established methods to derive single terrain attributes, if available. Given the circular nature of aspect, we used sine and cosine transformations to derive eastness and northness. Overall, we calculated 58 terrain attributes (Table 1) with SAGA GIS 2.3.1 [53].

**Table 1:** Terrain attributes used for SOC and bulk density modelling.

	Covariates	Method	Author(s)
Local	Slope and aspect	Fitted 2^nd^ degree polynomial	[54]
		Fitted 3^rd^ degree polynomial	[55]
		Least squares fitted plane	[56]
		Maximum triangle slope	[57]
		Fitted 2^nd^ degree polynomial	[58]
	Plan, profile, longitudinal, tangential and flowline curvature	Fitted 2^nd^ degree polynomial	[54]
		Fitted 3^rd^ degree polynomial	[55]
		Fitted 2^nd^ degree polynomial	[58]
	Vertical distance to channel network		[53]
	Sky visibility, sky view factor, direct and diffusive insolation		[59]
Regional	Catchment area	Top-down	[60]
		Recursive	
Combined	Topographic Wetness Index (TWI)	Any combination of slope and catchment area	[61]
	Slope length and steepness factor (LS-Factor)	Any combination of slope and catchment area	[61,62]

Terrain attributes derived from a DEM with a given resolution may not be suitable for landscape characterization and for digital soil mapping due to a non-representative DEM resolution [63], since the terrain attributes are not derived on the most relevant scale [64,65]. To examine the influence of scale, [65] applied simple smoothing (mean) filters with different neighbourhood sizes. This approach was applied on every terrain attribute used in this study with five circular neighbourhoods (radii of 1, 2, 4, 6 and 8 pixels), resulting in 290 terrain attributes in total. The maximum radius was set to 8 pixels to represent the local catena scale of 90 m.

### Machine learning techniques

We compared three data mining methods to test the 3D prediction of soil profile depth functions for SOC and BD based on terrain covariates. Given the large number of 290 covariates (instances) and sample sizes of n = 67 and n = 55, not all available techniques could be applied. For example, the interpretable multiple linear regression (MLR) analysis used for spatial modelling of polynomial depth functions by Aldana Jague [34] requires more samples (n) than instances (p; [66]). Furthermore, we have to account for multi-collinearity. Many terrain covariates in this study are calculated by different algorithms for the same terrain attribute and on different spatial scales with the same algorithm, which is often seen as a constraint in machine learning [66]. To reduce the covariate space to either enable MLR or handle the ‘curse of dimensionality’, principal component analysis (PCA) is often applied. However, feature reduction with PCA can have negative effects on model accuracy with multi-scale terrain data and models with the full set of covariates have higher accuracies [65]. Other feature reduction methods increase accuracy only marginally [65]. In this study, we applied multivariate adaptive regression splines (MARS), random forests (RF) and support vector machine (SVM). These machine learning methods are robust against multi-collinearity, can handle n<p [66] and select the most informative covariates without expert knowledge. Further, we omitted feature reduction.

For modelling, R version 3.3.1 was used [67]. For accessing the machine learning packages, the uniform interface *caret* [68] was used, which also offers data handling and model validation methods.

#### Multivariate adaptive regression splines (MARS)

MARS was introduced by Friedman [69] and is a generalisation of recursive partitioning regression approaches using piecewise linear models. With its linear basis functions, it overcomes the discontinuous response of other recursive partitioning models like Classification and Regression Trees (CART; [70]) and can generate continuous surfaces. Therefore, prediction accuracy of MARS is expected to be higher [69]. MARS is a partial linear function, where each new part is added with an exhaustive search for best fit and models a finite quantity of the regression. Thus, the model measures variable importance by its nature and is insensitive to non-informative instances. MARS require very little pre-processing and are non-affected by collinearity, since the predictor selection is random during iteration and redundant features are used equally [66]. This may affect measurement of variable importance and interpretation, which, however, is out of scope in this study. For modelling using MARS, the *earth* package version 4.4.6 [71] was used.

#### Random forests (RF)

RF is a widely used machine learning technique in digital soil mapping [17,22,64,72]. It was introduced by Breiman [73] and is an ensemble technique with CART [70] as a base learner. The single decision tree uses binary splits to create more homogenous groups in respect to the response. To grow an ensemble of trees, different random subsets of covariates (bootstrap sampling) and features (random set of features for every split) are used to build a single tree. The final prediction is created by averaging all individual tree outputs. Breiman [73] has proven that random forests with a large number of trees is robust against overfitting. Moreover, it is robust against noise, non-informative and correlated features. RF also returns feature importance measures (affected by correlation as MARS; [66]) and there is little need for fine-tuning [74]. The *randomForest* package version 4.6–12 [75] was used for modelling with RF.

#### Support vector machine (SVM)

Originally, SVM has been developed for classification problems [76]. It is a kernel method and uses hyperplanes to linearly separate classes of objects. For regression problems, Drucker et al. [77] developed support vector regression machines (SVR), which are an extension of SVM. Therefore, the term SVM is often used in both cases. The kernel function defines a transformation of the input data into a high dimensional feature space. In this feature space, it is possible to derive a linear regression hyperplane for non-linear relationships. Afterwards, it is back-transformed to non-linear space. Smola and Schölköpf [78] provide a comprehensive and detailed insight into SVR. The kernel used in this study is a radial basis function, where the scaling parameter σ is estimated by *caret* after a method by Caputo et al. [79]. In contrast to MARS, Drucker et al. [77] suggest that SVM should be used when the number of features is larger than the number of instances, since its optimisation does not depend on the dimensionality of feature space. Furthermore, SVM is partially insensitive to outliers (depending on cost factor) and does not require feature reduction to reduce multi-collinearity [66]. The *kernlab* package version 0.9–25 [80] was used for radial support vector regression modelling.

#### Data pre-processing

Some algorithms are sensitive to the scale and the range of the covariate space (e.g. SVM). To reduce effects of small values and little variance, SVM needs centred and scaled covariates [66], which was computed using the scale and centre-option in *caret*. To make all models comparable, this was also done for MARS and RF.

### Spatial 2.5D and 3D models

#### Differences between 2.5D models and spatial prediction of depth functions

The environmental covariates were used to train regression models (MARS, RF and SVM) to predict SOC and BD. For 2.5D predictions this was done for each sampled depth increment individually, were we assigned the mid-depth of the sampled increments as depth of the respective layer. This method to obtain volumetric soil information has several advantages. For modelling of each standard depth individually, there are no further requirements to abstract soil information in terms of vertical variability, i.e. a soil profile function. Furthermore, there is no error propagation through secondary models that describe depth functions. On the other hand, in contrast to 3D modelling, 2.5D modelling has the disadvantage that the individual model outcomes are purely two-dimensional. Soil properties of the depth increments between the standard depths are not used in the model and have to be derived on an interpretative basis [15] or through further processing [30] after spatial prediction. However, this is a well-established and well-documented approach. Therefore, we compare the results of the 3D approach described below directly with the 2.5D results.

#### 3D mapping with soil depth functions

**Fig 4:**
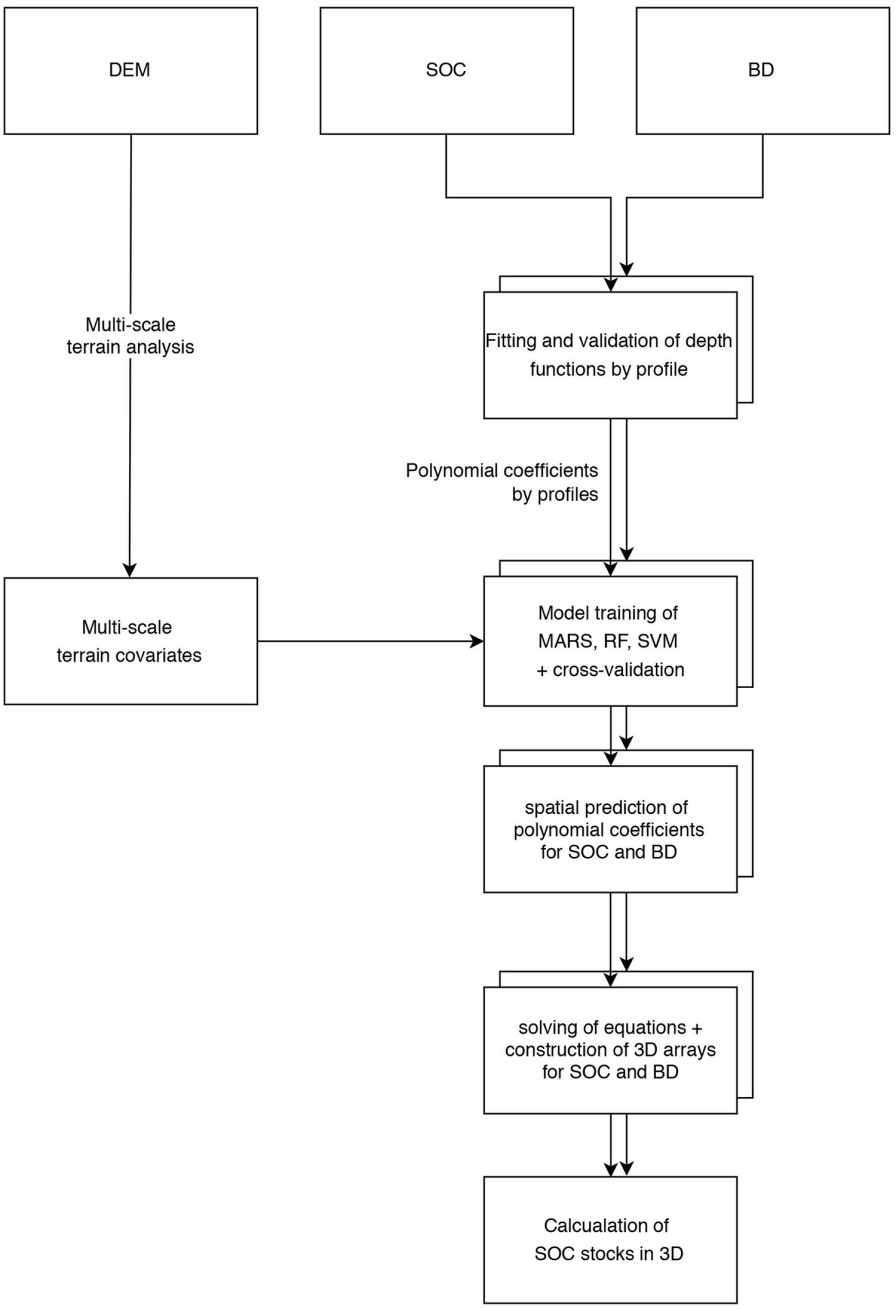
Flow chart summarizing the methodology steps of the 3D mapping and the used datasets at each step.

For the spatial modelling of depth functions, which we handled similar to the soil properties in terms of modelling, we applied 3^rd^ degree polynomial functions proposed by Aldana Jague [34] and less flexible 2^nd^ degree polynomials as well as logarithmic and exponential functions [32]. The workflow of the 3D mapping (Fig 4) of this study involved five main steps:

i) Mathematical approximation of depth functions to the five depth increments with a linear least squares approach. These were
  $$f_{1}(x)=c_{0}+c_{1}x+c_{2}x2+c_{3}x^{3}$$ (1)
  [34]
  $$f_{2}(x)={c_{0}+c}_{1}x+c_{2}x^{2}$$ (2)
  $$f_{3}(x)=c_{1}*ln(c_{2}x)$$ (3)
  [32]
  $$f_{4}(x)={exp}^{c_{1}+c_{2}x}$$ (4)
  cf. [32]
  where f_1,2,3,4_(x) is SOC and BD at a specific depth x (depth of the lower corner of a voxel in cm), c_0_ is the intercept that equals SOC and BD at depth 0 (cm) and the function coefficients c_1_, c_2_ and c_3_ are dimensionless. This altogether described the vertical distribution of SOC in respect to depth x at a certain location.
ii) Evaluation of model error for all equations in (i).
iii) Spatial modelling of the function coefficients c_1_, c_2_, c_3_ and c_0_ (analogous to two-dimensional modelling of SOC and BD) of the depth function with the lowest error (ii) with MARS, RF and SVM. The depth function parameters were treated and evaluated similar to a soil property.
iv) Evaluation of the cross-validation results for MARS, RF and SVM models of the depth function coefficients.
v) Solving the depth functions with spatially modelled coefficients (iii) at each grid location to generate a three-dimensional model.

The depth functions were solved for depths from 0 cm to 50 cm in 5 cm increments. The resulting 11 depth layers (matrices) were stacked to two three-dimensional models (one for SOC and BD each), where individual values are represented by voxels, which are the volumetric 3D analogue of 2D pixels. Due to the nature of the polynomial depth functions, negative SOC predictions in the profiles are possible. Consequently, the values of these voxels had to be set to zero. This is not required for logarithmic and exponential functions.

Compared to the standard depth method, the main advantages of spatially modelled depth functions are a higher vertical resolution and the fact that the result can be interpreted as volumetric structure. Instead of pixels with SOC and BD information in multiple layers, volumetric elements–so called voxels–in a three-dimensionally georeferenced stack of matrices with user-defined vertical resolution are obtained. Since the depth functions are secondary models, the error which is propagated by the depth function model to the spatial model depends on the chosen function. Due to the limited number of samples per profile, cross-validation of the depth functions was omitted.

The final models for SOC and BD were validated internally against the measured values of the input datasets.

### Validation and evaluation

The evaluation consists of two independent steps for the 2.5D multi-layered model predictions and the volumetric 3D model predictions of SOC and BD, where we treat the depth function parameters as soil properties.

In a first step, we evaluated each model of the soil properties SOC and BD as well as the spatial models of the depth function parameters, by using a 10-fold cross-validation with the coefficient of determination (R^2^) and the root mean square error (RMSE) as quality criterion. In this step, the models were tuned over the default grid- or hyper-learning sequence of parameters [81] using the tune grid function of *caret* to identify the most suitable combination of tuning parameters with the lowest RMSE and to reduce the model error, while preserving the models ability to generalise. The tuning parameters are *degree* and *nprune* for MARS, *mtry* for RF and *cost* for SVM. For RF *ntree* was set to the default value and σ for SVM was calculated by a method after Caputo et al. [79]. All models used the same set of folds to make cross-validation results comparable. The final models were selected from this sequence by the lowest RMSE.

To estimate the effect of overfitting of the depth function models based on grid learning, we evaluated the 3D model results with the datasets SOC and BD by R^2^ and RMSE (observed-predicted-evaluation). Overfitting is indicated by large differences in the prediction error between the training and the validation sets [81].

Further, we compared the 3D models against the 2.5D predictions of the same datasets to evaluate the performance of the 3D models. We chose this approach, because the legacy datasets are too small to hold out a larger subset for independent validation. The model results should be similar, if the spatial prediction of depth function parameters is reproducing the spatial distribution of the soil properties. This means that independently from the modelling framework (modelling of SOC and BD or modelling depth function as soil property) the results of the 3D model are reasonable, if both models are similar.

We see this comparison as a valid method for the evaluation of the 3D models, since Brus et al. [38] report strong correspondence between 2.5D and 3D geostatistical models and MARS, RF and SVM are well established for 2D and 2.5D soil mapping and in data science [17,27,66]. Therefore, we use the 2.5D layered predictions at the specific mid-depth of the increments as reference predictions. For the comparison between the 2.5D models and the corresponding depths of the 3D models, we used the coefficient of determination R^2^, Lin’s concordance correlation coefficient (ρ_c_; [82]), which validates the models against the 1:1 line, and the RMSE.

### Estimation of SOC stocks

The three-dimensional array of SOC stocks was calculated by

$${SOC}_{stocks}=\frac{SOC}{100}*BD*500^{2}*5$$ (5)

where SOC_stocks_ (g voxel^-1^) is the soil organic carbon storage, SOC is SOC content (%), BD is bulk density (g cm^-3^), 500^2^ is the base area of a voxel (cm^2^) related to the DEM resolution of 500 cm and 5 is the vertical resolution in cm. Consequently, 1 voxel represented 1.25 m^3^ of soil. Adjustment with the fraction of coarse material (> 2 mm) was omitted, since the coarse fraction was negligible low (< 5 vol.-%) at the VIPs and cannot be determined precisely by coring. According to Orton et al. [51] calculating the SOC stocks from two models of SOC and BD is an useful alternative when the samples are not taken at the same locations.

## Results

### 2.5D predictions of standard depths as reference

For the models of SOC, the mean cross-validation R^2^ of MARS was 0.33 with a root mean square error of 0.39%, compared to RF with an R^2^ of 0.41 (RMSE 0.34%) and SVM with an R^2^ of 0.39 (RMSE 0.35%; cf. Table 2). Models for BD showed a mean R^2^ of 0.43 (MARS), 0.39 (RF) and 0.39 (SVM) and mean RMSE values of 0.09 g cm^-3^ (MARS), 0.08 g cm^-3^ (RF) and 0.08 g cm^-3^ (SVM). In addition to the mean values, Table 2 shows the prediction accuracies and the RMSE’s for each depth increment and all three machine learning techniques of both SOC and BD.

**Table 2:** Performance of 10-fold cross-validation for MARS, RF and SVM applied on the sampled standard depths of SOC and BD.

		R^2^			RMSE		
	depth (cm)	MARS	RF	SVM	MARS	RF	SVM
SOC (%)	0–5	0.28	0.41	0.37	0.59	0.48	0.51
	0–10	0.25	0.41	0.42	0.46	0.4	0.4
	10–20	0.31	0.31	0.26	0.37	0.32	0.34
	20–30	0.46	0.47	0.46	0.3	0.28	0.29
	30–50	0.38	0.45	0.43	0.24	0.2	0.21
	$\bar{X}$	0.34	0.41	0.39	0.39	0.34	0.35
BD (g cm^-^³)	0–5	0.51	0.53	0.61	0.07	0.06	0.06
	0–10	0.5	0.52	0.49	0.07	0.06	0.06
	10–20	0.31	0.26	0.24	0.11	0.11	0.11
	20–30	0.41	0.35	0.33	0.1	0.1	0.1
	30–50	0.42	0.31	0.3	0.09	0.09	0.09
	$\bar{X}$	0.43	0.39	0.39	0.09	0.08	0.08

### Soil depth functions

For SOC, all equations showed R^2^ values higher than 0.9 (0.99 for f_1_, 0.96 for f_2_, 0.96 for f_3_ and 0.94 for f_4_) with a RMSE ranging from 0.36 (f_1_) to 0.7% (f_2_). For BD, the performance in terms of R^2^ was similar (RMSE = 0.07 g cm^-3^), except for f_3_ with R^2^ = 0.84 (RMSE = 0.22 g cm^-3^), which is the natural logarithmic function. The 3^rd^ degree polynomial (f_1_) resulted in the best fits for SOC and BD. However, the general trend of SOC in the profiles was exponential (Fig 2). Hence, both the 3^rd^ degree polynomial and the exponential functions were chosen for further spatial modelling and comparison in this study. With higher errors and without being able to reproduce the general trend in the profiles profile the 2^nd^ order polynomial (f_2_) was omitted in the following steps.

### Spatial modelling of soil depth functions

The cross-validation results for the machine learning methods applied on the depth functions (c.f. Table 3) showed, that the polynomial depth functions for MARS, RF and SVM for SOC were comparable in their goodness of fit with marginal differences (mean R^2^ from 0.3 to 0.32). R^2^ of the exponential depth functions ranged from 0.3 for MARS to 0.44 for RF.

**Table 3:** Performance of a 10-fold cross-validation for MARS, RF and SVM applied on function coefficients of a 3^rd^ degree polynomial (f_1_ for SOC and BD with four coefficients) and natural logarithmic function (f_3_ for BD with two coefficients).

		R^2^			RMSE			nRMSE		
		MARS	RF	SVM	MARS	RF	SVM	MARS	RF	SVM
SOC (f_1_)	c_0_	0.29	0.28	0.26	0.83	0.75	0.79	0.20	0.18	0.19
	c_1_	0.36	0.43	0.46	0.15	0.13	0.14	0.22	0.19	0.20
	c_2_	0.29	0.28	0.24	0.008	0.007	0.007	0.2	0.18	0.18
	c_3_	0.3	0.21	0.31	0.0001	0.0001	0.0001	0.14	0.14	0.14
	$\bar{X}$	0.31	0.3	0.32	-	-	-	0.19	0.17	0.18
BD (f_1_)	c_0_	0.56	0.45	0.38	0.09	0.09	0.09	0.23	0.2	0.20
	c_1_	0.38	0.34	0.218	0.02	0.02	0.02	0.14	0.14	0.14
	c_2_	0.38	0.17	0.26	0.001	0.001	0.001	0.2	0.2	0.2
	c_3_	0.25	0.27	0.31	0.00002	0.00002	0.00002	1.3×10^5^	1.2×10^5^	1.2×10^5^
	$\bar{X}$	0.39	0.31	0.28	-	-	-	3.2×10^4^	3×10^4^	3×10^4^
BD (f_3_)	c_1_	0.56	0.48	0.53	0.09	0.09	0.09	0.2	0.18	0.18
	c_2_	0.34	0.24	0.2	0.03	0.04	0.04	0.14	0.19	0.14
	$\bar{X}$	0.45	0.36	0.36	-	-	-	0.17	0.19	0.16

Note that coefficients dimensions are different and specifying a mean of the RMSE is not reasonable.

The models of the function coefficients could not be compared directly because c_0_ represented the SOC in % and BD in g cm^-3^, whereas c_1_, c_2_ and c_3_ were dimensionless. Hence, we compared these models by the normalised RMSE (nRMSE), which is the RMSE divided by the coefficients range (Table 3). The nRMSE showed little variation of around 0.18 for all coefficient predictions of the 3^rd^ polynomial depth function of SOC. RF had the lowest mean of nRMSE over all coefficients (0.17). The lowest nRMSE (0.09) for SOC was achieved by the exponential depth functions (RF and SVM).

The models based on the 3^rd^ degree polynomial depth functions of BD had a mean R^2^ of about 0.23–0.4, while the mean nRMSE was about 3×10^4^, due to the low performance of models with c_3_. Given such high errors, none of the models could reasonably predict the 3^rd^ degree polynomial depth function for bulk density. The exponential function was not able to reproduce the vertical trend of BD. Thus, we used the logarithmic depth function, although it fitted the five depth increments least. However, these spatial depth function models performed better (mean R^2^ from 0.36 to 0.45; nRMSE of about 0.16 for SVM).

### Evaluation of 3D predictions

For the comparison of 3D models against the 2.5D reference predictions, we used the coefficient of determination R^2^, Lin’s concordance correlation coefficient ρ_c_ and the RMSE in corresponding depths (Table 4).

**Table 4:** Coefficient of correlation (R^2^), Lin’s concordance correlation coefficient (ρ_c_) and RMSE of 2.5D reference predictions and correspondent depths of 3D predictions with polynomial (f_1_), logarithmic (f_3_) and exponential (f_4_) depth function.

		R^2^			ρ_c_			RMSE		
		MARS	RF	SVM	MARS	RF	SVM	MARS	RF	SVM
SOC (%; f_1_)	2.5 cm	0.02	0.92	0.89	-0.03	0.95	0.79	4.13	0.09	0.12
	7.5 cm	0	0.69	0.89	-0.01	0.81	0.85	3.84	0.16	0.1
	15 cm	0.02	0.41	0.72	-0.03	0.43	0.47	2.42	0.37	0.22
	25 cm	0	0.17	0.45	0	0.17	0.66	9.09	0.68	0.15
	40 cm	0	0.07	0.15	0	0.04	0.31	46.96	1.73	0.33
	$\bar{X}$	0.01	0.45	0.62	-0.01	0.48	0.62	13.29	0.61	0.18
SOC (%; f_4_)	2.5 cm	0	0.96	0.93	0	0.93	0.79	19.22	0.11	0.14
	7.5 cm	0.1	0.84	0.67	0	0.39	0.29	26.25	0.38	0.35
	15 cm	0	0.89	0.88	0	0.94	0.93	29.42	0.06	0.05
	25 cm	0.06	0.85	0.93	0	0.55	0.79	31.28	0.21	0.1
	40 cm	0.02	0.88	0.75	0	0.31	0.26	32.71	0.31	0.3
	$\bar{X}$	0.04	0.88	0.83	0	0.62	0.61	27.78	0.21	0.19
BD (g cm^-3^; f_3_)	2.5 cm	0.02	0.94	0.87	-0.05	0.39	0.53	0.48	0.09	0.07
	7.5 cm	0	0.8	0.71	0	0.29	0.24	0.44	0.09	0.1
	15 cm	0.01	0.66	0.5	-0.05	0.48	0.31	0.46	0.05	0.07
	25 cm	0.01	0.44	0.57	0.02	0.59	0.53	0.43	0.04	0.04
	40 cm	0.02	0.76	0.43	-0.12	0.17	0.08	0.64	0.1	0.11
	$\bar{X}$	0.01	0.72	0.62	-0.04	0.38	0.34	0.49	0.07	0.08

**Fig 5:**
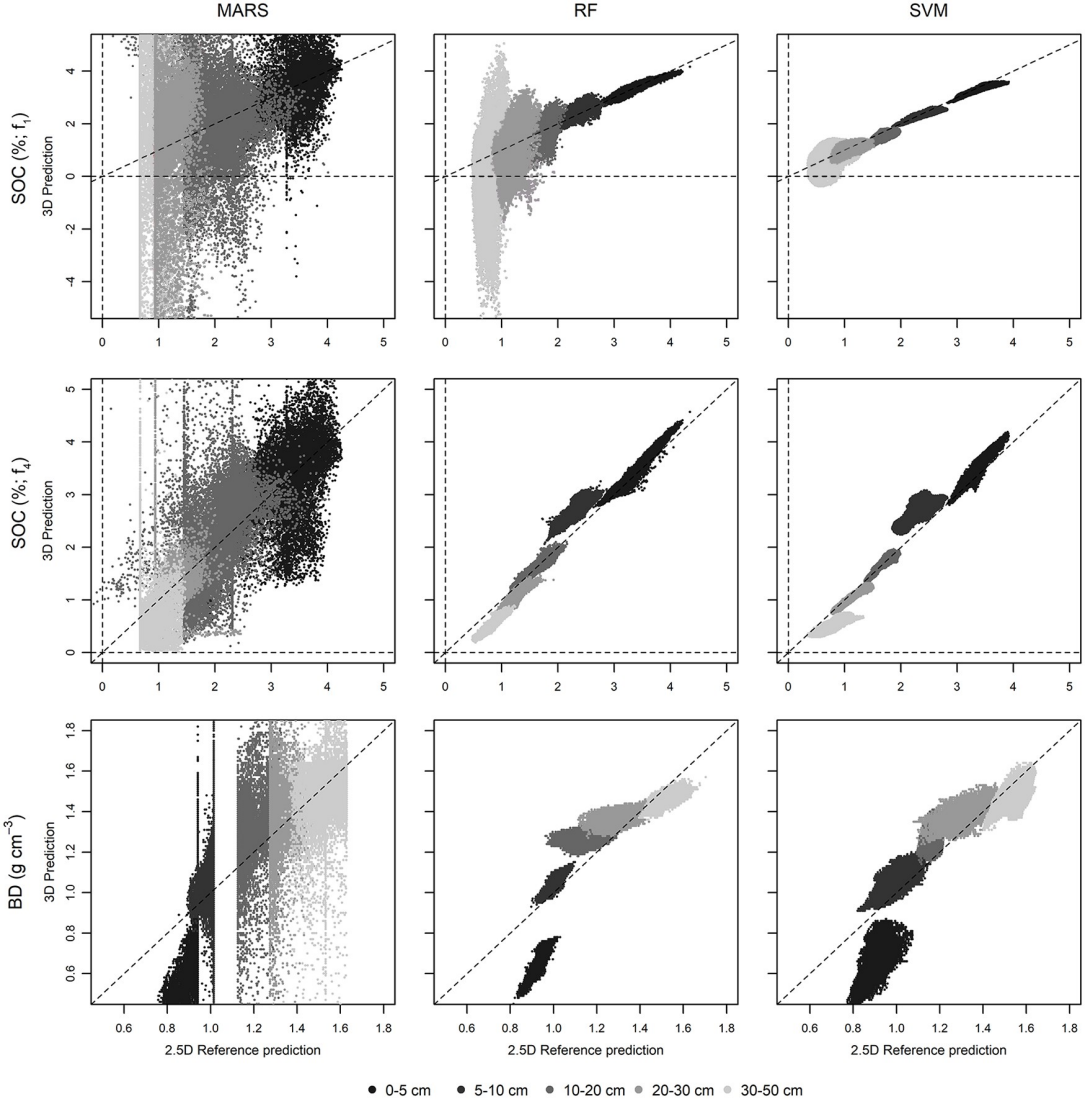
3D predictions of sampled depth increments plotted against corresponding 2.5D predictions. 3D prediction of SOC was calculated with 3^rd^ degree polynomials (upper row) and exponential function (middle row). The 3D prediction for BD with logarithmic function (lower row).

The three-dimensional MARS prediction for SOC with the 3^rd^ degree polynomial depth function showed the largest difference to its counterpart. The prediction at 2.5 cm ranged from close to zero to 15% SOC compared to 1.5 to 4% SOC in the two-dimensional prediction (Fig 5). The other depth increments showed a similar pattern with values down to -15% SOC. For the 2.5 cm increment the performance of RF was slightly better than that of SVM, but subsequently dropped with increasing depth. Especially at 40 cm, but also at 25 cm and 15 cm, the three-dimensional prediction of RF differed more from the two-dimensional predictions than the three-dimensional predictions of SVM differed from their counterparts. There was no distinct over- or underestimation of RF, but random scattering between -4 and 4% SOC for 40 cm (Fig 5). SVM showed lower deviation at 15 cm, 25 cm and even 40 cm. There were less predictions with negative values and less scattering. The predicted depth intersections of spatially modelled depth functions corresponded to the two-dimensional predictions by SVM largely by R^2^ and ρ_c_, while RMSE is low (Table 4).

In contrast, the 3D predictions of RF and SVM based on the exponential function showed good correspondence for all five depth increments (Table 4). The 3D predictions overestimated SOC for the 0–5 and 5–10 cm increments and underestimated it for 20–30 and 30–50 cm slightly due to the exponential nature of the equation, but there was no wide scattering as it was the case with the polynomial prediction for RF.

The results of the internal validation showed high correspondence between the chosen models (RF with exponential function for SOC and RF with logarithmic function for BD) and respective input data at all five sampled depth increments (Table 5). The R^2^ and RMSE values of the internal validation were similar to the validation results of the model comparison, indicating that model overfitting of both models is similar (Table 4). This partly accounts to the propagation error of the profile depth function. The spatial prediction of the exponential function for SOC had an average R^2^ of 0.79 with an average RMSE of 0.33% and the prediction of the logarithmic function used for BD had a R^2^ of 0.77 with an average RMSE of 0.14 g cm^-3^.

**Table 5:** Internal validation results of the final 3D models with the exponential function for SOC and the logarithmic function for BD.

	SOC (%)		BD (g cm^-3^)	
	R^2^	RMSE	R^2^	RMSE
2.5 cm	0.88	0.32	0.87	0.29
7.5 cm	0.74	0.47	0.85	0.07
15 cm	0.77	0.24	0.72	0.12
25 cm	0.76	0.29	0.74	0.08
40 cm	0.8	0.31	0.66	0.12
$\bar{X}$	0.79	0.33	0.77	0.14

### SOC stocks

The 2.5D models showed SOC stocks of 61.9 Mg ha^-1^ from 0 to 40 cm, with 19, 14.7, 12, 8.9 and 7.3 Mg ha^-1^ in the individual depth increments (from surface downwards).

**Fig 6:**
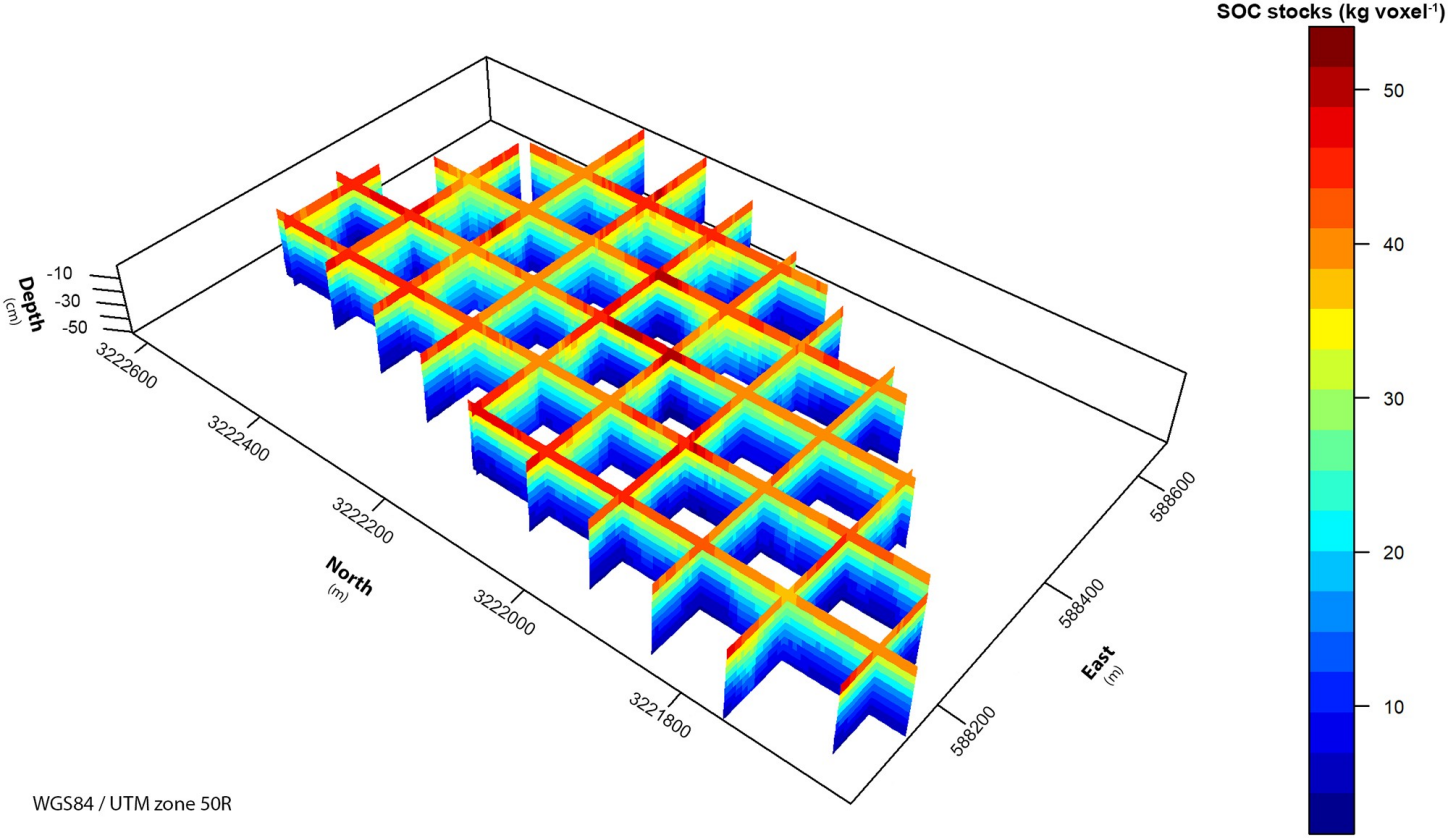
Three-dimensional prediction of SOC stocks for the whole catchment. The final 3D SOC stock model is shown in vertical slices 150 m apart to display the vertical variability, which is larger than the spatial variability.

The 3D model predicted 78.3 Mg ha^-1^ over the whole interval. The upper 20 cm of soil contained about 46.4 Mg ha^-1^. This depth is often designated as topsoil [83,84] and is also a critical soil depth for modelling plant productivity and community assembly [85]. 31.9 Mg ha^-1^ SOC are stored in the subsoil from 20 to 40 cm. Considering that the rooting depth varies, depending on the species and individual age, a static discrimination between topsoil and subsoil may be not appropriate. The model showed that plants with shallow roots down to 5 cm mainly interacted with a carbon pool of 10.9 Mg ha^-1^, whereas plants with roots in 25 cm depth interacted with a pool of 54.5 Mg ha^-1^. Fig 6 shows the 3D prediction of SOC stocks as vertical intersections of the solum. The highest stocks in the upper 5 cm were predicted in the central upper slopes and at the western slopes. Predictions for this depth at the valley bottom were around 20% lower. However, at the valley bottom the predictions for intermediate depth increments (around 30 cm) were higher than predictions at the upslope positions. The depth function for SOC stocks was much steeper and the SOC stock decline with depth was more pronounced at upslope positions compared to downslope and valley positions.

## Discussion

### 2.5D predictions of standard depths as reference

As RF returned the lowest error for the 2.5D models, this was the best choice for modelling SOC. SVM ranked slightly below. Compared to the results presented by Lacoste et al. [30], who used Cubist for 2.5D SOC stock mapping, the accuracy of our results was similar and reasonable.

However, the sampled VIPs do not represent the terrain of the study site adequately, since they were chosen based on species richness levels, which were distributed randomly, and not representative for the study site. For example, a representative sampling design could be achieved with Conditioned Latin Hypercube Sampling (cLHS) [72,86].

For bulk density SVM and RF performed equal by means of R^2^ and RMSE and showed a similar pattern, especially at 15 cm and 40 cm. MARS performed least for BD. In general, RF resulted in the most stable predictions and is therefore recommended over SVM.

### Evaluation of 3D predictions

The negative values in the prediction results and the pronounced difference between the 3D models, with predictions up to 15% SOC, and the 2.5D models indicated that MARS is not capable of adequately predicting the depth functions in space, although the cross-validation showed similar results as for RF and SVM models. The latter showed better correspondence between the 3D and the 2.5D models (Fig 5, Table 4). According to the results of the direct comparison between the multi-layered prediction and the corresponding depths in the 3D model, RF with exponential functions was most suitable for SOC modelling. RF and SVM with polynomials performed well at upper depth increments and less in lower increments. MARS models were not suitable of reproducing the 2.5D predictions. Lower performance of all techniques with polynomials in the lower depth increments may be referred to lower influence of the terrain as a driving factor to explain SOC accumulation and redistribution (e.g. by erosion). Other factors accounting for SOC redistribution in deeper soil horizons may be bioturbation or vertical transport in the liquid soil phase. Additionally, it is possible, that accumulation layers in the solum, that would reflect the lateral distribution, were not fully covered by the legacy dataset and, therefore, the interpretation remains difficult. All these processes and others relevant for SOC concentration as well as SOC stocks cannot be fully covered by a distinct set of terrain parameter and lead to a dilution effect by predicting the deeper horizons. Lower accordance of the models also may be referred to uncertain models of function coefficients (c_3_) and (c_2_), which have significant influence at greater depths (cubic and squared) and exponentiate up this error. Based on the results, we chose RF with exponential depth functions for three-dimensional mapping of SOC and the logarithmic depth function for BD.

### SOC stocks

Compared to other studies in this area, the estimated SOC stocks were well in line. Scholten et al. [19] calculated mean SOC stocks of 70 Mg ha^-1^ for the upper 50 cm with the same data but a different approach. Chen et al. [83] compared five plantations with different species in five age groups and calculated SOC stocks for the upper 20 cm. Especially the age of the trees and shrubs and their biomass have a strong impact on SOC stocks. Very young forest communities showed SOC stocks ranging from 20 to 25 Mg ha^-1^ and plantations with older trees of 7 to 10 years 30–40 Mg ha^-1^. The latter were slightly older than the trees of BEF-China, where 42 Mg ha^-1^ were predicted. Diverse species pools in these studies may explain differences. Tang et al. [87] found SOC stocks in the top 60 cm in bamboo forests ranging from 60 to 200 Mg ha^-1^.

The introduced approach is capable of summing SOC stocks at any depth interval. Since topsoil depth varies spatially, conventional static assumptions of topsoil thickness can result in inaccurate SOC stock calculations for individual horizons. Incorporating spatial models of topsoil depth into 3D SOC stock mapping can overcome this drawback and help to improve ecological and biodiversity models as conducted in the BEF-China experiment. In particular, consideration of biotic predictors like forest biomass, tree species richness and functional plant diversity might further improve model fit and accuracy of estimated SOC stocks [14]. This would allow one to quantify terrain-specific effects of changes in forest cover and composition on SOC stocks. The developed models could also help to identify areas that are especially prone to loss of SOC stocks (e.g. by soil erosion or land cover change).

Furthermore, continuous three-dimensional SOC mapping can support models of a national SOC inventory. Yang et al. [37] applied depth functions to categorical soil types and estimated SOC stocks for mainland China. Combining models with high vertical resolution by Yang et al. [37] and continuous spatial modelling like in this study can improve accuracy of SOC mapping compared to the categorical mapping approach. This combination can also help to estimate and understand carbon fluxes between topsoil and subsoil [88] as well as between soil and the atmosphere [5]. Both objectives play major roles in inventory estimation, SOC auditing and decision making in respect to ecosystem services and carbon sequestration [1,5,7,12,89].

## Conclusion

This study comprises the spatial prediction of soil depth functions for three-dimensional modelling of SOC and bulk density. The spatial prediction of the function coefficients enabled the calculation of two three-dimensional arrays by solving the depth functions for depths from 0 to 50 cm by 5 cm increments. This was used to estimate the SOC stocks in high spatial (5 m) and vertical (5 cm) resolution. The main conclusions of this study are:

- The general trend of SOC as visualised by the boxplots (Fig 2) was exponential. However, polynomial depth functions described the soil profiles for SOC with higher accuracy and the logarithmic functions for BD showed better results in spatial modelling. Therefore, we conclude that functions resulting in high accuracies based on the soil profile data may not be the most suitable for spatial modelling, as they may overfit the vertical trend of SOC content.
- The 3D RF models correspond best with the 2.5D counterparts (R^2^ up to 0.96). Thus, RF is recommended to predict SOC based on exponential depth functions and bulk density with logarithmic depth functions in high vertical resolution. The 2.5D and 3D predictions of SOC with RF correlated much better, especially when using exponential functions, and lacked accuracy in deeper layers for SOC when modelled based on polynomial functions.
- Comparisons between conventional 2D and 2.5D predictions at the sampled depth and the corresponding depth of the three-dimensional predictions showed that MARS is not suitable for modelling corresponding 2.5D and 3D models, although cross-validation of the individual models showed similar performance in R^2^.

Minor conclusions are: polynomial functions may be an option, when the problem of propagated errors and the ability to generalise in the horizontal domain is investigated further, however, polynomials of any degree have to be used carefully. To overcome these shortcomings, a higher sampling density in the vertical and horizontal domain and in combination with other depth functions, such as equal-area splines [90], should be considered, since exponential functions are not suitable for soil properties that do not increase or decrease continuously.

The 3D approach presented in this study is promising for SOC auditing in various disciplines and especially for decision making regarding climate and land use policies. Future work should focus on sampling design to cover valley positions outside the established plots at site A of BEF-China project. Given the dynamics of SOC stocks, we recommend the analyses of time series data and the expansion of the current database for four-dimensional models.
